# Characterization of Different Functionalized Lipidic Nanocapsules as Potential Drug Carriers

**DOI:** 10.3390/ijms13022405

**Published:** 2012-02-22

**Authors:** Paola Sánchez-Moreno, Juan Luis Ortega-Vinuesa, Antonio Martín-Rodríguez, Houría Boulaiz, Juan Antonio Marchal-Corrales, José Manuel Peula-García

**Affiliations:** 1 Biocolloid and Fluid Physics Group, Department of Applied Physics, University of Granada, 18071 Granada, Spain; 2 Human Anatomy and Embryology Department, Regenerative Biomedicine Institute (IBIMER), Campus de la Salud, University of Granada, 18071 Granada, Spain; 3 Department of Applied Physics II, University of Málaga, 29071 Málaga, Spain

**Keywords:** nanocarriers, lipid nanocapsules, immuno-nanocapsules, drug delivery

## Abstract

Lipid nanocapsules (LNC) based on a core-shell structure consisting of an oil-filled core with a surrounding polymer layer are known to be promising vehicles for the delivery of hydrophobic drugs in the new therapeutic strategies in anti-cancer treatments. The present work has been designed as basic research about different LNC systems. We have synthesized—and physico-chemically characterized—three different LNC systems in which the core was constituted by olive oil and the shell by different phospholipids (phosphatidyl-serine or lecithin) and other biocompatible molecules such as Pluronic^®^ F68 or chitosan. It is notable that the olive-oil-phosphatidyl-serine LCN is a novel formulation presented in this work and was designed to generate an enriched carboxylic surface. This carboxylic layer is meant to link specific antibodies, which could facilitate the specific nanocapsule uptake by cancer cells. This is why nanoparticles with phosphatidyl-serine in their shell have also been used in this work to form immuno-nanocapsules containing a polyclonal IgG against a model antigen (C-reactive protein) covalently bounded by means of a simple and reproducible carbodiimide method. An immunological study was made to verify that these IgG-LNC complexes showed the expected specific immune response. Finally, a preliminary *in vitro* study was performed by culturing a breast-carcinoma cell line (MCF-7) with Nile-Red-loaded LNC. We found that these cancer cells take up the fluorescent Nile- Red molecule in a process dependent on the surface properties of the nanocarriers.

## 1. Introduction

Nanomedicine, an emerging new field created by the fusion of nanotechnology and medicine, has become one of the most promising pathways for developing effective targeted therapies with particular impact on oncology. This is because these new therapeutic strategies could supply the tools necessary to improve inherent limitations of classical pharmacotherapy [1–4]. In recent years, different colloidal systems such as nanoparticles and nanocapsules have been reported as potential carriers for drug delivery [5,6]. Structured nanocapsules are generally described as colloidal systems with a core-shell structure [1,2], where the core acts as a liquid reservoir for several molecules or drugs, and the shell as a protective membrane. These nanosystems present promising applications as carriers of drugs, proteins or DNA chains, or in diagnosis applications as contrast agents [7,8]. Their useful properties include biocompatibility and biodegradability, low toxicity, controlled release of drugs and the ability to target specific tissues [5]. In particular, lipid nanocapsules (LNC), consisting of an oil-filled core with a surrounding polymer shell have special use for encapsulating and delivering hydrophobic drugs. The versatility of these LNC for an efficient encapsulation in their oily core of several anti-cancer drugs has been previously demonstrated [9,10]. It should be noted that, due to the hydrophobic character of many of these drugs, current pharmacotherapy must use solubilizer agents for their intravenous administration [11], which in turn is another foreign substance that has to be added in the blood stream. This is why LNC offer a promising system and an excellent alternative to emulsions or microemulsions for pharmaceutical application of hydrophobic drugs [7,12,13]. Thanks to the drug protection and their controlled release on cancer cells, these kinds of nanoparticles provide an ideal solution, leading to selective cytotoxicity, minimizing the serious and unpleasant side effects of cancer drugs and preventing damage to healthy tissues [14,15].

The shell of LNC can be formed by a wide variety of polymers/surfactants with hydrophilic segments such as polyethylene glycol (PEG), polyethylene oxide, poloxamers, poloxamines, polysorbates, chitosan, *etc*. [16–19]. These types of polymers, in addition to enhancing the intrinsic colloidal stability of the system, may help to avoid their recognition by the mononuclear phagocyte system (MPS), a major drawback that often arises after intravenous injection of drug carriers, causing a decrease in circulating LNC as well as undesirable accumulation of these colloids in the liver or the spleen. Therefore, the nature of the polymeric shell is crucial to minimize the MPS action by repelling plasma proteins [16] and achieving the so-called Stealth^®^ nanosystems [5,11,20–22]. This would increase the *in vivo* long-term stability of the nanoparticles, and it would also facilitate their ability to cross certain biological barriers. For example, nanoparticles coated with polysorbates or poloxamers have been reported to successfully pass the blood-brain-barrier and other physiological barriers [23].

On the other hand, although LNC represent an important class of nanocarriers capable of efficiently encapsulating and delivering a variety of drugs, their typical pathway to act on cancer tissues is through the so-called enhanced permeability and retention effect. This means passive targeting with non-specific delivery and the inability to cross several biological barriers based on molecular recognition processes [7,10,15]. Thus, it would be advisable to improve the efficacy of chemotherapy as well as to decrease the systemic toxicity of these drugs by using tailor-made tumor-targeted drug carriers, thereby reducing—although not completely avoiding—unspecific passive delivery. Vectorization and targeting capacities of these systems can be implemented by surface modification with specific biomolecules (e.g., antibody fragments, folic acid) conjugated to LNC and enhancing the cell-targeting through molecular recognition processes such as ligand-receptor or antigen-antibody reactions [6,10,24–26]. Today, it is possible to form a LNC surface with several agents bearing diverse functional groups capable of covalently binding a variety of biochemically active groups. Shell polymers are generally synthesized with pendant functional groups such as hydroxyl, carboxyl, amine or thiol groups (−OH, −COOH, −NH_2_, or −SH). As a result, these tailored-LNCs would deliver a given drug specifically towards a targeted malignant tumor [4].

Within this scenario, the main objective of the present work focuses on developing a simple, reproducible and non-expensive procedure to synthesize LNCs systems, paying special attention to designing nanocapsules in which antibody molecules can be covalently attached on the surface. Thus, several lipidic nanosystems with different surface characteristics have been obtained and analyzed in order to acquire a fuller knowledge concerning the physicochemical properties of these colloidal particles, closely examining the role played by the components. Thus, a thorough characterization was made, including size, electrokinetic behavior, and colloidal stability. Specifically, we have synthesized three different core-shell lipid nanosystems by using a simple process with commercially available biocompatible components. In all cases, the hydrophobic core was constituted by olive oil, while the hydrophilic shell nature was varied by adding different molecules in order to generate different (and desirable) surface properties. The molecules used in the surface modification were phospholipidic molecules, a poloxamer, and chitosan. Thus, we have two typical reference systems previously reported [18] with an anionic and a cationic surface charge respectively, and a novel nanosystem (not described in the literature yet) in which the shell was constituted by phosphatidyl-serine and a poloxamer producing a carboxyl-functionalized nanosystem. In the second step, we developed the chemical immobilization of a classical polyclonal IgG antibody on the carboxylated nanocapsules by means of a reproducible and simple method. For this, a well-established procedure based on the carbodiimide (CDI) method was used [27–30]. All the antibody-LNC systems were physico-chemically characterized and compared with bare LNC. The immunological response of our colloidal immune-nanocapsules was also quantified against the specific ligand of the antibody molecules, the C-reactive protein (CRP). The goal of this part was to check whether this new LNC system enriched by phosphatidyl-serine could efficiently link antibodies for future vectorization purposes, and whether these antibodies kept their intrinsic immuno-reactivity once they were immobilized on the LNC surface.

The work finishes with an *in vitro* study to evaluate the potential use of our LNC. In this last part, Nile-Red-loaded lipid nanocapsules were prepared to make a quantitative study of particle uptake by a breast-carcinoma cell line (MCF-7), Nile-Red being a commercially available hydrophobic fluorescent molecule.

## 2. Results and Discussions

**Figure 1:**
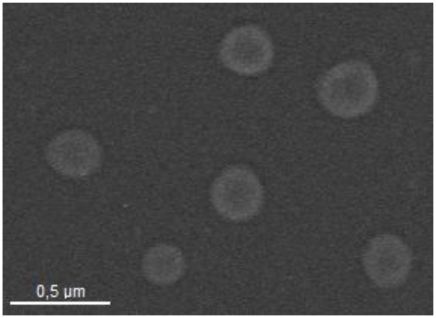
Transmission electron microscopy photography of the EPI nanocapsules.

The first part of this work includes a complete physicochemical and morphological characterization of our colloidal systems. Their synthesis procedures are shown in experimental section. The shell of the simplest system (referred to as **EPI**) was constituted by a commercial mixture of phospholipid molecules (Epikuron) and a poloxamer (Pluronic^®^ F68), both acting as colloidal stabilizers. A second system (referred to as **CS**) was formulated by substituting the poloxamer by chitosan oligomers. Finally, in the third formulation Epikuron was substituted by phosphatidyl-serine (referred to as **pHS**). Particle size and size distribution are key variables to determine the *in vivo* distribution of the nanoparticles, the drug release, the targeting ability, and their colloidal stability [31,32]. Generally, a sub-micron size is recommended in the literature. It is advisable to formulate nanoparticles with an optimal size depending on their specific use, normally under 200–300 nm of diameter, and preserving, at the same time, the colloidal stability of the system [16]. In our case, the synthesis procedure yielded to spherical nanocapsules with an average diameter in the nanometric scale, Table 1. It should be noted that the size of all the formulations remained at a constant value under the storage conditions (pure water, 4 °C) for at least four months, which is a good indication of their intrinsic stability in water. The morphology of the nanocarriers was analyzed by TEM. The three LNC systems showed a spherical shape and a size consistent with the values previously found using light back-scattering measurements. Figure 1 shows a TEM micrograph of the EPI nanocapsules.

Given the size differences among the EPI and PhS nanoparticles, the “epikuron-poloxamer” pair appears to be a better emulsifier than the “PhS-poloxamer” one, in that the former produces particles with a smaller diameter than the latter. Previous results have shown that the presence of poloxamer together with lecithin increases the particle size in comparison to the case in which lecithin was the only component of the shell [18]. This feature could be extrapolated to the PhS nanoparticles (with a larger diameters) suggesting a greater incorporation of poloxamer when this surfactant is added together with phosphatidyl-serine, producing nanoparticles with a larger diameter. Results of electrokinetic measurements and colloidal stability (shown in the following sections) appear to support this idea. Nevertheless, the most significant result was the size increment found when chitosan was added together with epikuron. In this case, positive chitosan chains can interact electrostatically with negative phospholipid molecules, reducing the effective concentration of this latter emulsifier and, consequently, producing larger nanocapsules [18].

With regard to the immuno-nanocapsules, we will firstly explain why our PhS-LNCs were sensitized with a partial coating of IgG. It is well known that the presence of polyclonal IgG molecules linked onto colloidal particles causes a significant alteration of their surface properties, changing the electrokinetic behavior and reducing significantly the colloidal stability at neutral pH [28]. The development of colloidally stable nanosystems is a prerequisite for successful future applications of drug carriers in biological media. Taking into account that a low stability has been found working with other nanoparticles of similar size as ours when their surfaces were saturated with IgG molecules (5 mgIgG/m^2^) [33], we developed systems with a low to medium IgG coverage: 1 mgIgG/m^2^, and 2.5 mgIgG/m^2^. The covalent coupling was developed by means of the CDI method, described in detail in the experimental section, and no aggregation was noted over the experimental period. After coupling, immuno-nanocapsules were separated from unbound protein by a dialysis procedure. An analysis of the eluted solution volume from dialysis by means of spectrophotometric measurements did not detect any presence of protein molecules in the solution, which would indicate very high coupling efficiency. The size of these antibody-nanocapsule complexes (at pH 8 and low-ionic-strength medium) is shown in Table 1. Diameters remain similar to those of the original PhS-system regardless of the antibody amount on the surface. This is a good indication that the appropriate selection was made with our antibody density values, since an increase in the hydrodynamic diameter has been reported for several nanosystems having a high density of antibodies immobilized on them, meaning low stability and a strong tendency to aggregate [25,34].

**Figure 2:**
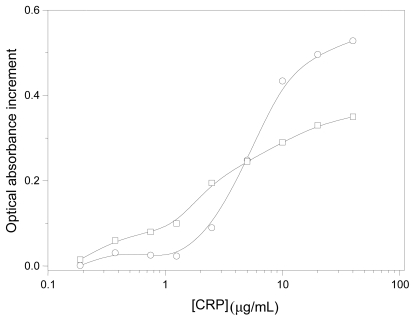
Absorbance change after 300 s (λ = 570 nm) due to aggregation induced by different C-reactive protein (CRP) concentrations: (○) PhS-1 mgIgG/m^2^; (□) PhS-2.5 mgIgG/m^2^. Reaction medium: 13 mM borate (pH 8.0), 150 mM NaCl, 1 mg/mL NaN_3_, 1 mg/mL BSA. The starting point of optical absorbance for all experiments was 0.5.

Prior any further physico-chemical characterization of the immuno-complexes, it was necessary to test whether the immobilized IgG maintained its antigenic activity. Previous results working with hard nanospheres in which IgG was covalently linked with the CDI method showed good immuno-activity [27,28]. However, we are now working with soft liquid particles where IgG might denature when located in the oil/water interface and/or may change the preferential orientation of the adhered IgG molecule—especially when surfactant molecules (e.g., poloxamer) are placed on the surface [35]. Therefore, we quantified the immuno-reactivity by spectrophotometrically measuring the agglutination extent when our immuno-nanocapsules were mixed with the corresponding antigen (CRP). These experiments are determinant to test the feasibility and potential applicability of these soft lipid immuno-nanocapsules in future active targeting strategies. Figure 2 shows the changes in the optical absorbance *vs.* the CRP concentration for both immuno-complexes. It should be noted that at low CRP concentrations, those particles with a higher IgG coating were more reactive, while at [CRP] <5 μg/mL the highest increment of absorbance was found with the 1 mgIgG/m^2^ complex. Nevertheless, the immuno-agglutination extent was significant in both cases and it indicates an adequate surface disposition of the antibody molecules for specific recognition.

**Figure 3:**
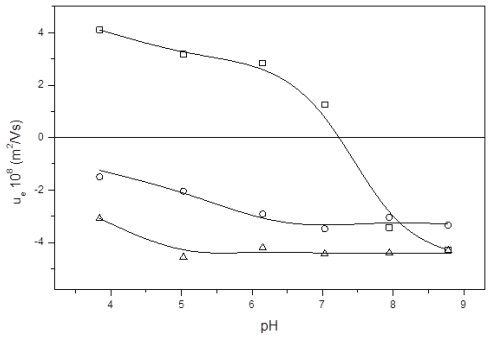
Electrophoretic mobility *vs.* pH for (▵) EPI; (□) CS; and (○) PhS nanocapsules.

The next set of experiments was focused on determining the electrical state of the nanocapsules at different pH values. A magnitude commonly used to gain information about the surface state of charge of colloidal particles is electrophoretic mobility (μ_e_). This is an experimental parameter directly related with the zeta potential existing in the shear plane of the particles [28,36]. The electrophoretic mobility data, gathered from low-ionic-strength media, are shown in Figure 3. The μ_e_ values depend on the electrical potential at the shear plane, which is ultimately governed by the composition of the LNC surface and by the salinity and pH conditions of the medium in which the particles are dispersed. The μ_e_ results agree with the nature of the shell of our nanocapsules and they confirm the presence of the different molecules used in their synthesis. That is, the EPI and PhS nanocapsules showed typical behavior of colloids with weak acid groups, giving lower μ_e_ values at acidic pH values than those found at neutral and basic pH. In addition, Figure 3 shows a peculiar nuance related with the nature of the surface-charged groups. These groups come exclusively from the Epikuron molecules in the EPI nanocapsules, in which phosphatidyl-choline is the major component. Thus, the phosphatidic acid—with a p*K*_a_ between 3 and 4—is the main charged group in these LNC. However, the PhS-nanoparticles present only phosphatidyl-serine as phospholipidic molecules; that is, in this case there are two different surface charged groups: phosphatidic and carboxylic (p*K*_a_ = 4.8). This is also reflected in the electrokinetic behavior, since the reduction in the mobility value begins at pH 5 for the PhS system while it is at pH 4 for the EPI particles (see Figure 3). This subtle difference in our experimental μ_e_ measurements agrees with other data gathered with similar colloidal systems formed by both phosphatidic [18] and carboxylic [37] surface groups. The presence of Pluronic^®^ F68 molecules on the surface does not alter the electrical state of the surface, since this poloxamer is a non-ionic surfactant [18,35]. However, a μ_e_ reduction (in absolute value) could be expected after the incorporation of this non-ionic surfactant onto the nanoparticle surface, since the presence of polyethylene oxide (PEO) chains would cause an outward shift of the shear plane where the ζ-potential is defined, and this would subsequently diminish the electrophoretic mobility. At least for polystyrene or polylactic-co-glycolic acid particles the μ_e_ reduction was significant and directly related to the poloxamer coating [35,38]. The results shown in Figure 3 for both EPI and PhS systems would indicate a higher incorporation of poloxamer for the PhS nanoparticles with an appreciable decrease of the absolute μ_e_ values throughout the pH range studied, as experimentally observed. As will be shown, the stability experiments corroborate this assumption related to the higher incorporation of Pluronic^®^ F68 in the PhS-LNC.

On the other hand, the μ_e_ behavior of the CS nanocapsules becomes radically different. These CS-LNC show mobility data very similar to those found with pure chitosan nanogels [39] and they practically coincide with previously results found for similar chitosan nanocapsules [18]. The μ_e_ confirms that the incorporation of chitosan was clearly effective when this polysaccharide was added to the formulation. It can be seen that mobility goes from positive values at acidic pH to large negative values at more basic pH, presenting an isoelectric point (i.e.p.) at pH 7. The positive charge of nanocapsules is provided by the glucosamine groups of chitosan, which present a weak basic character. At basic pH, chitosan chains are uncharged, so that the negative μ_e_ comes from the lecithin phosphatidic groups. This μ_e_ behavior indicates a clear incorporation of this polycationic polymer into the nanocapsule shell yielding to a surface structure practically formed (in its outer part) by a chitosan layer.

**Figure 4:**
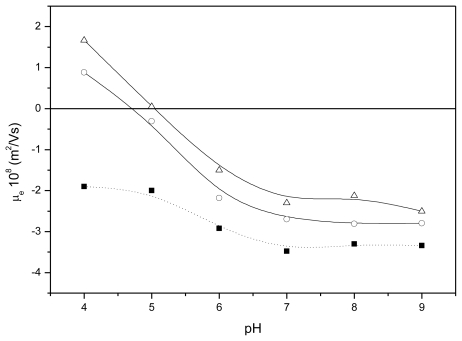
Electrophoretic mobility *vs.* pH for (■) PhS; (○) PhS-1 mgIgG/m^2^; and (▵) PhS-2.5 mgIgG/m^2^ IgG.

Usually, the presence of IgG molecules on the surface of colloidal particles causes a significant alteration of the μ_e_ values in comparison with the same bare surfaces. Therefore, the electrokinetic behavior of our immuno-nanocapsules can also be useful to determine the presence of such active material (IgG) linked on the LNC surface. Figure 4 shows the mobility of our immuno-nanocapsules as a function of the medium pH. These data support the presence of antibody molecules in the particle surface. Also, a clear correlation is found between the mobility data and the amount of surface protein. It is known that, when colloidal particles are coated by a protein, the original isoelectric point of bare particles moves towards the isoelectric point of the pure protein. In the case of our immuno-capsules, they inverted the original PhS-LNC μ_e_ sign at acidic pH 4 (due to the positive electrical charge of IgG molecules at these pH values [28,37,40]), shifting the isoelectric points of the complexes at pH 4.6 and 5.1 for the 1 mgIgG/m^2^ and 2.5 mgIgG/m^2^ systems, respectively [41,42]. On the other hand, analyzing the μ_e_ values at neutral and basic pH, we found another important difference among bare nanocapsules and immuno-nanocapsules, with a clear μ_e_ decrease (in absolute value) when the antibody coating increased. In these conditions, the surface charge density of the surface protein layer became significantly low, which translated as a reduction of the original negative surface electrostatic potential. It is worth remembering that μ_e_ values are usually indicative of the colloidal stability of the particles. Therefore, a reduction in μ_e_ values at neutral and basic pH values would imply decreased colloidal stability. As shown below, these complexes present low colloidal stability at physiological pH.

In the next set of experiments, the colloidal stability was studied at a pH value which matched that of culture media (7.4) used in the *in vitro* experiments in order to analyze the stability/instability of the nanocapsules when incubated with cells. Aggregations were induced by salinity using NaCl and CaCl_2_ independently. The corresponding CCC and CSC values for all the systems–nanocapsules and immuno-nanocapsules—are shown in Table 2. With regard to the CCC data, calcium exerts a much higher destabilizing effect than does sodium, as expected. This is due to the double valence of calcium, which screens the nanocapsule surface charge much better than sodium does. Consequently, the CCC values are consistently lower for CaCl_2_ than for NaCl.

Next, we examine the results found for bare nanocapsules. The CCC and CSC data gathered for the EPI nanocapsules are similar to those previously obtained for nanocapsules formulated with the same shell composition [18]. The incorporation of poloxamer into the shell confers a more hydrophilic character ascribed to the PEO fragments. The CSC data corresponding to CaCl_2_ confirm the presence of poloxamer molecules on the surface, showing a restabilization process typical of surfaces with hydrophilic character. This stability observed at moderate and high ionic strengths is governed by repulsive hydration forces that are explained in detail in references [43,44]. With regard to the NaCl, the EPI sample became completely stable in the entire NaCl-concentration range. This is due to the fact that the CCC is higher than the CSC, overlapping both critical concentrations in this highly hydrophilic system. That is, the restabilization mechanism based on hydration forces begins to act at a salt concentration in which the classical DLVO potential barrier still has not been definitively eliminated by the screening effect of the electrolyte concentration [18].

When we analyzed the colloidal stability of PhS nanocapsules, a completely stable system was found, since it was impossible to coagulate at any NaCl or CaCl_2_ salt concentration. This feature, characteristic of sterically stabilized colloids, clearly indicates a higher incorporation of Pluronic^®^ F68 molecules into the PhS system in contrast to the EPI case. Furthermore, this result agrees with previous electrokinetic data, in which a reduction in μ_e_ values has been related to the incorporation of PEO chains on the surface shell, which (consequently) shifted the shear plane where the zeta potential is defined outward.

Performing the experimental study of the stability of the CS system was a difficult task, because these LNC coagulated—even before adding any salt concentration—as soon as the nanocapsules were immersed in the buffer at pH 7.4. This high instability at neutral pH is consistent with the isoelectric point shown in the μ_e_ experiments (see Figure 3), and it indicates that repulsive electric forces are the main factor responsible for the stability of the CS system at low ionic strength. Nevertheless, our deacetylated chitosan is a hydrophilic material, and therefore repulsive hydration forces should be expected to act under high-salinity conditions. This is why we immersed the CS particles in a pH 7.4 buffer but previously adding a moderate or high NaCl concentration. It is worth highlighting that this saline-system was totally stable. This is why the CSC data shown in Table 2 for the CS-LNC were gathered from a high-salinity solution and making successive dilutions that reduced the ionic strength and, consequently, triggered coagulation at a given low-salinity value.

In summary, the stability patterns of our systems comes from a combination of the destabilizing power exerted by salinity (which screens the electrical repulsion existing in low-ionic-strength media), and the stabilizing effect given by hydration forces in hydrophilic surfaces when salinity increases. When the electrolyte concentration is not too high (and hydration forces can be neglected), the CCC values correlate with the μ_e_ data in the less hydrophilic systems (EPI and CS), the stability being governed mainly by the surface electrical charge (which in turn depends on the nature of the shell molecules). For high-salinity conditions or for highly hydrophilic surfaces (*i.e.*, PhS-LNC) both repulsive steric and hydration forces keep stable the systems.

**Figure 5:**
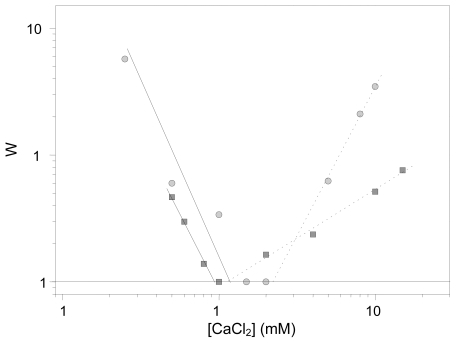
Stability factor *vs.* calcium chloride concentration (mM) at pH 7.4: (■) 1 mg/m^2^ IgG-PhS; (●) 2.5 mg/m^2^ IgG-PhS. Solid lines help to locate the CCC values, while dashed lines point to the CSC data.

With regard to the colloidal stability of our immuno-complexes, Figure 5 shows a typical experiment in which the stability factor (W) is evaluated at different CaCl_2_ concentrations. The W-stability factor is an experimental parameter that provides information on the coagulation probability: it is related to the number of collisions that two colliding particles must undergo before they remain definitively stuck. Therefore, W = 1 signifies a completely unstable system, while W = ∞ means total stability. The corresponding CCC values (calculated when W reduces to 1) are given in Table 2. These CCC data correlate properly with the mobility data at neutral pH (see Figure 3). The stability decreased when the IgG coating was increased. Although both systems presented quite low stability (CCC with CaCl_2_ ~10 mM), it was even lower when the IgG load was higher. It should be noted that the total stability found with the 1 mgIgG/m^2^ system in NaCl solutions disappeared when the IgG coating increased up to 2.5 mgIgG/m^2^. There is no doubt that the stability behavior of the PhS system significantly changed when the IgG molecules were linked to the bare PhS nanoparticles: A completely stable system in both salt solutions reduces its colloidal stability when protein molecules are bounded on its surface. This phenomenon has been also found when working with other nanoparticles-protein complexes totally or partially covered by polyclonal IgG molecules [28,33,45]. Beduneau *et al*., working with lipid nanocapsules conjugated with antibody molecules, detected an aggregation of the immuno-nanoparticles with a high density of antibodies [10], while Koning *et al*. detected the same stability when a high density of antibodies was grafted onto liposomes [34].

**Figure 6:**
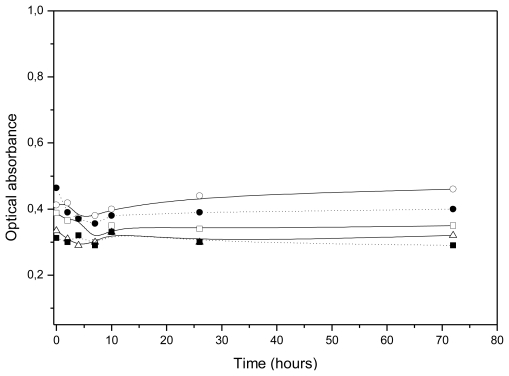
Aggregation kinetics of the (□) EPI; (○) CS; (▵) PhS; (■) PhS-1 mg/m^2^ IgG; and (●) PhS-2.5 mg/m^2^ IgG nanocapsules when immersed in DMEM medium supplemented with fetal bovine serum (FBS).

Once the stability was evaluated in simple pH 7.4 media, it was analyzed in the cell-culture medium (DMEM) supplemented with FBS, used to develop the *in vitro* uptake experiments in order to ensure that our different nanosystems preserve their colloidal stability in this complex medium. Figure 6 shows the time evolution of optical absorbance when nanocapsules were introduced in the cell-culture medium. The absorbance remained constant, indicating that no aggregation took place. Taking into account the CSC values shown in Table 2, the high stability of all our LNC systems in this culture medium must be governed by the intrinsic hydrophilic character of their respective surfaces, since DMEM is a medium enriched with different cations that strengthen hydration forces enough to keep all of our nanoparticles stable.

**Figure 7:**
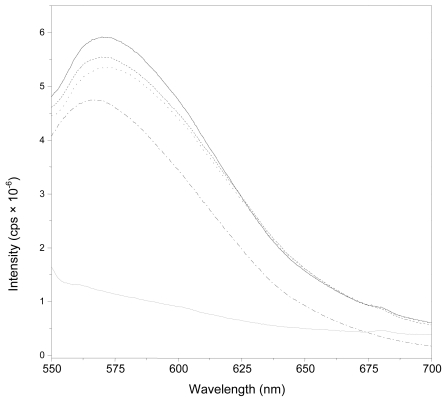
Emission spectrum of Red Nile in olive oil and water, and inside different nanoparticles. (black line) EPI; (dashed line) CS; (dotted line) PhS; (dash-dot line) Olive oil; (gray line) water.

The final experiments were focused on analyzing the uptake of our different nanocapsules by a cancer-cell line (MCF-7). To quantify this cell uptake, we worked with nanocapsules loaded by Nile Red. The presence of Nile Red in the oily core of our nanoparticles was previously checked by means of fluorescent measurements. It should be noted that Nile Red presents a very different emission fluorescent spectrum as a function of the medium in which it is dissolved [46]. Figure 7 shows the emission fluorescent spectrum of the different nanoparticle systems. For all of them, the spectrum was similar to those found for Nile Red dissolved in olive oil. Comparing these results with the Nile Red emission spectrum dissolved in an aqueous solution, we found that the presence of this fluorescent molecule in the hydrophobic oil core of our LCN was, therefore, clearly confirmed. On the other hand, as expected, the encapsulation of Nile Red hardly changed the size or charge of LNC.

**Figure 8:**
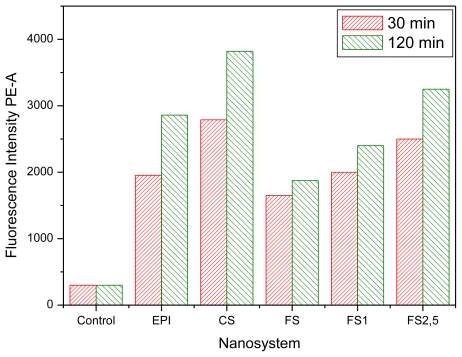
Relative fluorescent intensity of the MCF7 cell line when incubated with Nile-Red-loaded nanocapsules for 30 min and 2 h. Control refers to that incubation performed only with cells.

The cell uptake of these fluorescent nanocapsules was analyzed by flow cytometric assays. Figure 8 shows the fluorescence intensity of the cell cultures containing different nanosystems after 30 min and 2 h of incubation time. The results led us to perform a quantitative analysis of the different nanocapsules, showing that the internalization efficacy was significantly changed by the nature and properties of the different shells. A combination of surface charge and hydrophilicity plays the major role in affinity in the endocytosis pathway [47]. Results with CS nanocapsules showed the highest fluorescent intensity, in agreement with the enhanced mucoadhesion properties reported for chitosan nanosystems [9,48]. The lower fluorescent response observed with the EPI and PhS systems appeared to be related both to the negative surface charge of these nanocapsules as well as to the hydrophilic character provided by poloxamer molecules—which produce more biocompatible systems but at the same time reducing their cellular uptake [10,49]. The higher hydrophilicity of the PhS surface (which presents an enriched Pluronic^®^ F68 layer according to the mobility and stability data) could be responsible for the low fluorescent intensity shown by these nanocapsules in comparison with the EPI ones.

Finally, Figure 8 also shows an increment of the fluorescence intensity when the IgG coverage increased. Since our aCRP-IgG molecules did not recognize any specific ligand of the cell membrane, a change in the cell uptake of immuno-nanocapsules with regard to the bare nanocapsules must have been caused by both the surface charge and hydrophilic characteristics, which in turn were modulated by the protein coating. Actually, the results agree with the surface characteristics discussed above for our immuno-nanocapsules, considering their electrophoretic and stability behavior. That is, the electric charge and hydrophilic character decreased when the amount of bounded IgG was increased, yielding to a situation in which the LNC endocytosis was enhanced.

## 3. Experimental Section

### 3.1. Reagents

Olive oil, poloxamer 188 (Pluronic^®^ F68), Nile Red and phosphatidyl-L-serine were obtained from Sigma Aldrich. The olive oil was purified with activated magnesium silicate (Florisil, Fluka) in order to eliminate free fatty acids. Epikuron 145 V, which is a deoiled, wax-like, phosphatidyl-choline-enriched fraction of soybean lecithin, was kindly provided by CargilIbérica SL. Protasan^®^ Cl 113, and medium-molecular-weight chitosan chloride salt with a deacetylation degree of 85%, was supplied from FMC Biopolymer Novamatrix (Norway). For the study of the electrophoretic mobility and colloidal stability at different pH values, several buffered solutions with a low ionic strength (I = 0.002 M) were prepared: pH 4 and 5 were buffered with acetate, pH 6 and 7 with phosphate, and pH 8 and 9 with borate. Additionally, in some cases, stability was evaluated in phosphate buffer saline (PBS) and different culture mediums: Dulbecco’s Modified Eagle’s Medium (DMEM) supplemented with fetal bovine serum. Other chemicals and electrolytes used were of analytical grade and purchased from Sigma, Merck, and Scharlau. Deionized Milli-Q water was used throughout.

### 3.2. Nanocapsule Preparation

**Figure 9:**
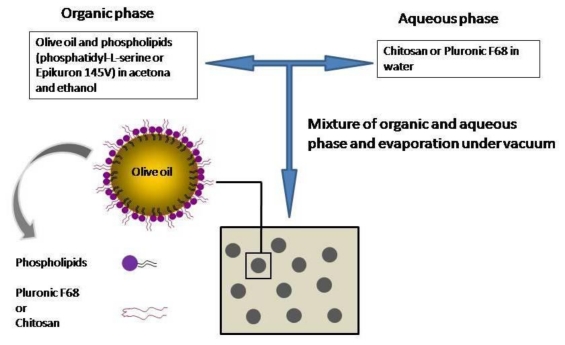
Scheme detailing the preparation of the particles.

The nanosystems studied were prepared by a solvent-displacement technique following the procedure of Prego *et al*. [50]. This is a well-known technique widely reported for the preparation of nanocapsules, where hydrophilic surfactants are usually dissolved in the aqueous phase before emulsion formation. Briefly, an organic phase was prepared containing 125 μL of olive oil and phospholipids: 40 mg of Epikuron 145 V (EPI nanocapsules) or 10 mg of phosphatidyl-L-serine (PhS nanocapsules) dissolved in 0.5 mL ethanol and 9.5 mL of acetone. This organic phase was immediately poured over an aqueous phase containing 50 mg of Pluronic^®^ F68 or 10 mg of chitosan oligomers. The mixture turned milky immediately because of the formation of the nanoemulsion. Then, the organic solvents were evaporated under vacuum to a final volume of 15 mL. Figure 9 shows a scheme of the synthesis procedure. All nanosystems presented olive oil in their hydrophobic core and, depending on the composition of the organic and aqueous phases, the final sample showed different interface properties. Thus, three different systems were formulated: **EPI nanocapsules** with a surface shell composed by epikuron and Pluronic^®^ F68; **CS nanocapsules** with epikuron and chitosan oligomers; and finally, **PhS nanocapsules** with phosphatidyl-L-serine and Pluronic^®^ F68.

Fluorescent LNCs were formulated by dissolving Nile Red in the olive oil phase at a concentration of 0.025% (w/w). The encapsulation of this fluorescent molecule was confirmed by using a SPEX Fluoromax-2 spectrofluorometer. The emission fluorescence spectra were determined from 550 to 700 nm previous excitation of the sample at a wavelength of 485 nm, with a scanning speed of 100 nm/min and a wavelength accuracy of ±1 nm.

### 3.3. Nanocapsule Characterization

#### 3.3.1. Size, Morphology, and Storage Stability

The average size of the nanosystems was determined by combining a high-performance-particle-sizer technology with a non-invasive-backscattering method, where the measurement is taken at an angle of 173°. This technique minimizes multiple scattering [51] and the light detected is processed by a digital correlator (ALV 5000/E), providing information on the average diffusion coefficient of the particles, which can be easily related to the mean diameter (Ø) by using the Stokes-Einstein equation for spheres. The morphological characterization of the nanosystems was developed using transmission electron microscopy (TEM) in the Scientific Instrumentation Centre, Granada University (Spain).

#### 3.3.2. Electrophoretic Mobility

A Zetasizer-nano (Malvern Instruments, UK) was used to measure the electrophoretic mobility (μ_e_). The study was focused on measuring the μ_e_ as a function of pH while maintaining a constant low-ionic-strength value (0.002 M). Each μ_e_ mobility datum was the average of 6 individual measurements.

#### 3.3.3. Colloidal Stability

Colloidal stability was spectrophotometrically studied (Beckman DU 7400 spectrophotometer) working at physiological pH in simple saline media. The salts used in these stability studies were NaCl and CaCl_2_. As described in detail in reference [52] and the supplementary materials, it is possible to derive the “critical coagulation concentration” (CCC) and the “critical stabilization concentration” (CSC) values. These parameters are fundamental in colloidal-stability studies, since they give valuable information about certain surface characteristics. For example, CCC is related to the surface charge density, while the CSC is associated to the surface hydrophilicity [51]. In addition to measuring the colloidal stability of our nanocapsules with NaCl and CaCl_2_, we also evaluated stability in PBS and in DMEM supplemented with fetal bovine serum by monitoring the variation of optical absorbance of the nanocapsules as a function of time.

### 3.4. Immuno-Nanocapsules

#### 3.4.1. Conjugation of IgG Antibodies with LNC

C-reactive protein (CRP) and polyclonal immunoglobulin G-anti-CRP (aCRP-IgG) from rabbit were obtained, purified, and kindly donated by Biokit S.A. (Spain). The covalent attachment of aCRP-IgG to the PhS nanoparticles was performed according to the well-established carbodiimide method [27]. In this method, 1 ethyl-3-(3-dimethyl aminopropyl) carbodiimide (CDI) was used to bind the antibody via its amino groups to the carboxyl groups of the PhS nanoparticles. Briefly, a sample of PhS system having 0.2 m^2^ of total surface was incubated at pH 8 with CDI (100 μL of a solution of 15 mg/mL) for 30 min at 25 °C. Subsequently, activated nanoparticles were incubated with two different amounts of aCRP-IgG, 1 mg/m^2^ and 2.5 mg/m^2^, overnight. All the immuno-nanocapsules were cleaned by means of a dialysis process in pH 8 buffer solution in order to separate the non-coupled aCRP-IgG molecules.

#### 3.4.2. Immunological Study

The immunoassays were performed in saline BSA borate buffer: pH 8, borate (15 mM), NaCl (150 mM), NaN_3_ as preservative (1 mg/mL), and bovine serum albumin from Sigma-Aldrich (1 mg/mL). The immuno-nanoparticles were diluted in this solution to provide a working LNC reagent. A series of CRP solutions was prepared in the saline BSA medium, the antigen concentration ranging from 0.25 μg/mL to 40 μg/mL. Turbidimetric curves of absorbance *vs.* time were plotted using a simple spectrophotometer (Beckman DU 7400). Absorbance measurements were collected at 570 nm for 300 s at 25 °C, after mixing 50 μL of each antigen solution with 150 μL of the immuno-LNC solution.

### 3.5. Cell Line and Culture Conditions

A breast-carcinoma cell line (MCF-7) was supplied by the Scientific Instrumentation Centre, Granada University (Spain). Cells were grown at 37 °C in an atmosphere containing 5% CO_2_, with DMEM (Gibco, Grand Island, NY, USA) supplemented with 10% (v/v) heat-inactivated fetal bovine serum (FBS), 2% L-glutamine, 2.7% sodium bicarbonate, 1% Hepes buffer, and 1% of penicillin/streptomycin solution. The intracellular uptake of fluorescence-labeled nanoparticles was performed as follows. MCF-7 cells (3 × 10^4^) were seeded into 6-well plates under the culture conditions detailed above. After 24 h, cells were fed with fresh medium and treated with Nile-Red-nanocapsules. After incubation for 30 min and 2 h with particles labeled with Red Nile the cells were washed with PBS to remove free nanocapsules. Then the cells were harvested by PBS-ethylenediamine-tetraacetid acid (PBS-EDTA), washed twice with cold PBS, and pelleted by centrifugation at 1500 rpm for 5 min. Living cells were resuspended in PBS and analyzed for red fluorescence by flow cytometry in a FACScan (Becton Dickinson, San Jose, CA, USA). The fluorescent pulses were analyzed by BD CellQuest Pro software [53]. Instrument settings, such as the photomultiplier voltage and compensation, were adjusted in such a way that the difference in background fluorescence of cells without internalized nanocapsules became insignificant. SPSS [54] was used for the statistical analysis in cell culture studies. The results were compared by means of the Student’s *t* test. Differences were considered statistically significant at a *p* value of <0.05.

## 4. Conclusions

The present work constitutes basic research aimed mainly at analyzing certain surface characteristics (*i.e.*, charge and hydrophilicity) of different lipid nanocapsules. Colloidal properties of a novel LNC system, in which the shell was enriched by carboxyl groups supplied by phosphatidyl-serine molecules, was compared to two standard LNC systems: an anionic one formed by Epikuron and Pluronic^®^ F68, and a cationic system coated by chitosan. Electrokinetic mobility and classical studies of colloidal stability lead us to conclude that the incorporation of Pluronic^®^ F68 on the PhS shell was higher than in the EPI case. Additionally, the original PhS surface was successfully modified by linking (covalently) IgG molecules. The presence of these antibodies conferred specific recognition properties to these nanocapsules, showing clear binding of a specific antigen (CRP). In addition, the adhered antibody layer altered the original surface properties of the bare PhS particles, since both surface charge density and hydrophilicity were reduced by the IgG coating. With regard to the cell-culture incubation studies, all our systems were colloidally stable in the incubation medium thanks to the stabilizing role played by the hydration forces. The cellular uptake was modulated by the surface characteristics of the LNC, and the highest uptake was found with the CS cationic particles, while the lowest intracellular incorporation was found with the most hydrophilic negative surface (PhS). In the latter case a great amount of poloxamer molecules existed in the outer shell, hampering such uptake. In summary, we have developed a novel LNC system capable of linking antibodies by using a simple procedure yielding to active immuno-nanocapsules that may be useful for future potential applications in anti-cancer therapies.

## Figures and Tables

**Table 1:** Particle size of the different lipid nanocapsule and immuno-nanocapsule systems.

Nanosystem	Diameter (nm)
**EPI**	170 ± 20
**CS**	340 ± 30
**PhS**	210 ± 20
**PhS-1 mgIgG/m** ^2^	200 ± 20
**PhS-2.5 mgIgG/m** ^2^	204 ± 14

**Table 2:** Critical coagulation concentration (CCC) and critical stabilization concentration (CSC) data (in mM units), at pH 7.4, using NaCl and CaCl_2_ as aggregating salts.

pH 7.4	EPI		CS		PhS		PhS-IgG (1 mgm-2)		PhS-IgG (2.5 mgm-2)	

	CCC	CSC	CCC	CSC	CCC	CSC	CCC	CSC	CCC	CSC
**NaCl**	stable	stable	aggreg	161	stable	stable	stable	stable	38	56
**CaCl** **_2_**	61	77	aggreg	14	stable	stable	12	22	9	12
